# Early-season crop mapping using improved artificial immune network (IAIN) and Sentinel data

**DOI:** 10.7717/peerj.5431

**Published:** 2018-08-31

**Authors:** Pengyu Hao, Huajun Tang, Zhongxin Chen, Zhengjia Liu

**Affiliations:** 1 Key Laboratory of Agricultural Remote Sensing, Institute of Agricultural Resources and Regional Planning, Chinese Academy of Agricultural Sciences, Beijing, China; 2 Key Laboratory for Geo-Environmental Monitoring of Coastal Zone of the National Administration of Surveying, Mapping and GeoInformation & Shenzhen Key Laboratory of Spatial Smart Sensing and Services, Shenzhen University, Shenzhen, China; 3 Institute of Geographic Sciences and Natural Resources Research, Chinese Academy of Sciences, Beijing, China

**Keywords:** Classification, Cotton, Maize, Early-season, Wheat, Hengshui, Sentinel, Image time series, Improved artificial immune network

## Abstract

Substantial efforts have been made to identify crop types by region, but few studies have been able to classify crops in early season, particularly in regions with heterogeneous cropping patterns. This is because image time series with both high spatial and temporal resolution contain a number of irregular time series, which cannot be identified by most existing classifiers. In this study, we firstly proposed an improved artificial immune network (IAIN), and tried to identify major crops in Hengshui, China at early season using IAIN classifier and short image time series. A time series of 15-day composited images was generated from 10 m spatial resolution Sentinel-1 and Sentinel-2 data. Near-infrared (NIR) band and normalized difference vegetation index (NDVI) were selected as optimal bands by pair-wise Jeffries–Matusita distances and Gini importance scores calculated from the random forest algorithm. When using IAIN to identify irregular time series, overall accuracy of winter wheat and summer crops were 99% and 98.55%, respectively. We then used the IAIN classifier and NIR and NDVI time series to identify major crops in the study region. Results showed that winter wheat could be identified 20 days before harvest, as both the producer’s accuracy (PA) and user’s accuracy (UA) values were higher than 95% when an April 1–May 15 time series was used. The PA and UA of cotton and spring maize were higher than 95% with image time series longer than April 1–August 15. As spring maize and cotton mature in late August and September–October, respectively, these two crops can be accurately mapped 4–6 weeks before harvest. In addition, summer maize could be accurately identified after August 15, more than one month before harvest. This study shows the potential of IAIN classifier for dealing with irregular time series and Sentinel-1 and Sentinel-2 image time series at early-season crop type mapping, which is useful for crop management.

## Introduction

The increase of global population increase has led to huge pressure on global food security (Alexander et al., 2015). As cropland distribution maps are the basement of the crop growth monitoring work and also important inputs of the crop production models, there is an urgent need of accurate in-season cropland mapping, particularly for major staple food crops such as wheat and maize (Wu et al., 2014).

Satellite observations have demonstrated good potential for identifying crop types. Multi-temporal data are most commonly used for crop type classification, since image time series can describe the phenological differences among different crops (Atzberger, 2013; Pan et al., 2011). However, there remain drawbacks to this technique. Generally, early-season crop type maps are important inputs for regional and global crop monitoring systems (Chen et al., 2011; Waldner et al., 2015). Most existing studies use image time series of the entire growing season to generate crop type maps; however, such maps are often acquired too late to inform crop management (Whitcraft, Becker-Reshef & Justice, 2015; Zhong et al., 2016). Furthermore, to identify crop types in the early season, there is a need of high temporal frequency image time series. Most high temporal frequency data are provided at coarse spatial resolution, such as EOS-MODIS and NOAA-AVHRR data, which can observe the land surface once daily (Van Leeuwen, Huete & Laing, 1999). Conversely, data with better spatial resolution has a lower temporal frequency, such as the most commonly used Landsat TM/ETM+/OLI data, which provides 30 m resolution images at a 16-day temporal frequency (NASA, 2003; USGS, 2013, 2017). Furthermore, the remote sensing data from moderate resolution optical satellites (such as Landsat) are further limited by missed observations due to cloud obscuring the Earth’s surface (Marshall, Dowdeswell & Rees, 1994). Therefore, moderate spatial resolution image time series are always irregular, which is a problem for early season crop type mapping. Although some gap-filling methods and high spatial and temporal resolution image fusion methods have been proposed to generate 30 m image time series with good temporal frequency (Gao et al., 2006; Wu et al., 2017; Zhu, Liu & Chen, 2012), the methods are generally time consuming and the results always have some uncertainty for crop type classification. In addition, these methods need to use images covering the entire growing season as input, which limits their ability for early season crop type mapping.

An alternative method is the image composition strategy, which uses the median value of all available pixels during one month as a composited value for each month. This method makes full use of the available observations and is simple to interpret. However, there is a contradiction between the period of composition and the information contained in the composited image time series. Long composition periods (e.g., 6 months) are less informative and cannot support crop type identification, while short composition periods (e.g., 15–30 days) lead to “missing value” pixels in the image time series (Van Leeuwen, Huete & Laing, 1999; Wolfe, Roy & Vermote, 1998; Xiong et al., 2017). Although various classifiers have been proposed for image classification, such as classification tree-based classifiers and machine learning classifiers, those methods have difficulty dealing with irregular image time series with missing values (Breiman et al., 2013; Howard & Wylie, 2014; Mountrakis, Im & Ogole, 2011; Waheed et al., 2006). Therefore, classifiers that can handle irregular time series are important for early season crop type mapping based on short period compositions of image time series (15–30 days or even shorter).

In addition, most existing crop type classification results were derived using image time series with coarse or moderate resolution, such as those of MODIS and Landsat. However, the relatively coarse spatial resolution results in many mixed pixels (pixels containing several crop types) in images of small fields (Lobell & Asner, 2004; Muchoney et al., 2000). In addition, the pixels at the crop field boundary may become mixtures of crops fields, roads and other land cover types. Thus, the mixed pixels cause spectrum variation and misclassification (Hao, Wang & Niu, 2015b). Several works have conducted crop type mapping using very high-resolution image time series, such as Gaofen-1, RapidEye and Quickbird images (Hao, Wang & Niu, 2015b; Turker & Ozdarici, 2011). However, the regions studied were generally small (<20 × 20 km), and the data are not freely available worldwide. As the Sentinel mission provides both C-Band SAR (Sentinel-1) data and optical (Sentinel-2) data at high spatial resolution (10 m) and high revisit frequency (6 days for Sentinel-1 and 5 days for Sentinel-2), it is worth investigating the potential of early season crop type mapping using Sentinel image time series (Drusch et al., 2012; ESA, 2015, 2016; Van Der Meer, Van Der Werff & Van Ruitenbeek, 2014).

In this study, we tried to identify crop types in the early season from 10 m resolution Sentinel-1 and Sentinel-2 data. The objectives were (1) to propose a method which can deal with missing values in satellite remote sensing data for crop type mapping and (2) to evaluate how early in the growing season accurate crop type maps can be generated.

## Study Area and Data Sets

**Figure 1:**
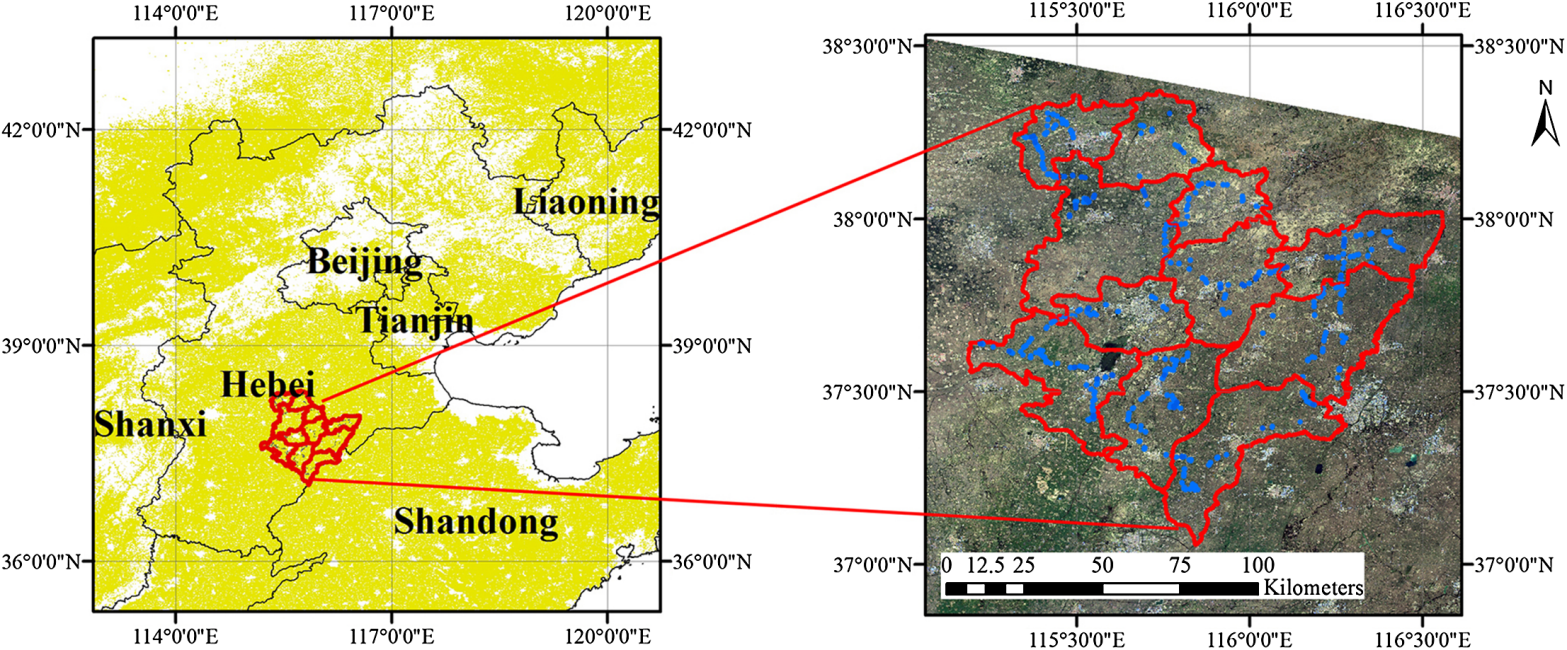
Study area. The blue dots in this figure represent the in situ surveyed fields.

**Figure 2:**
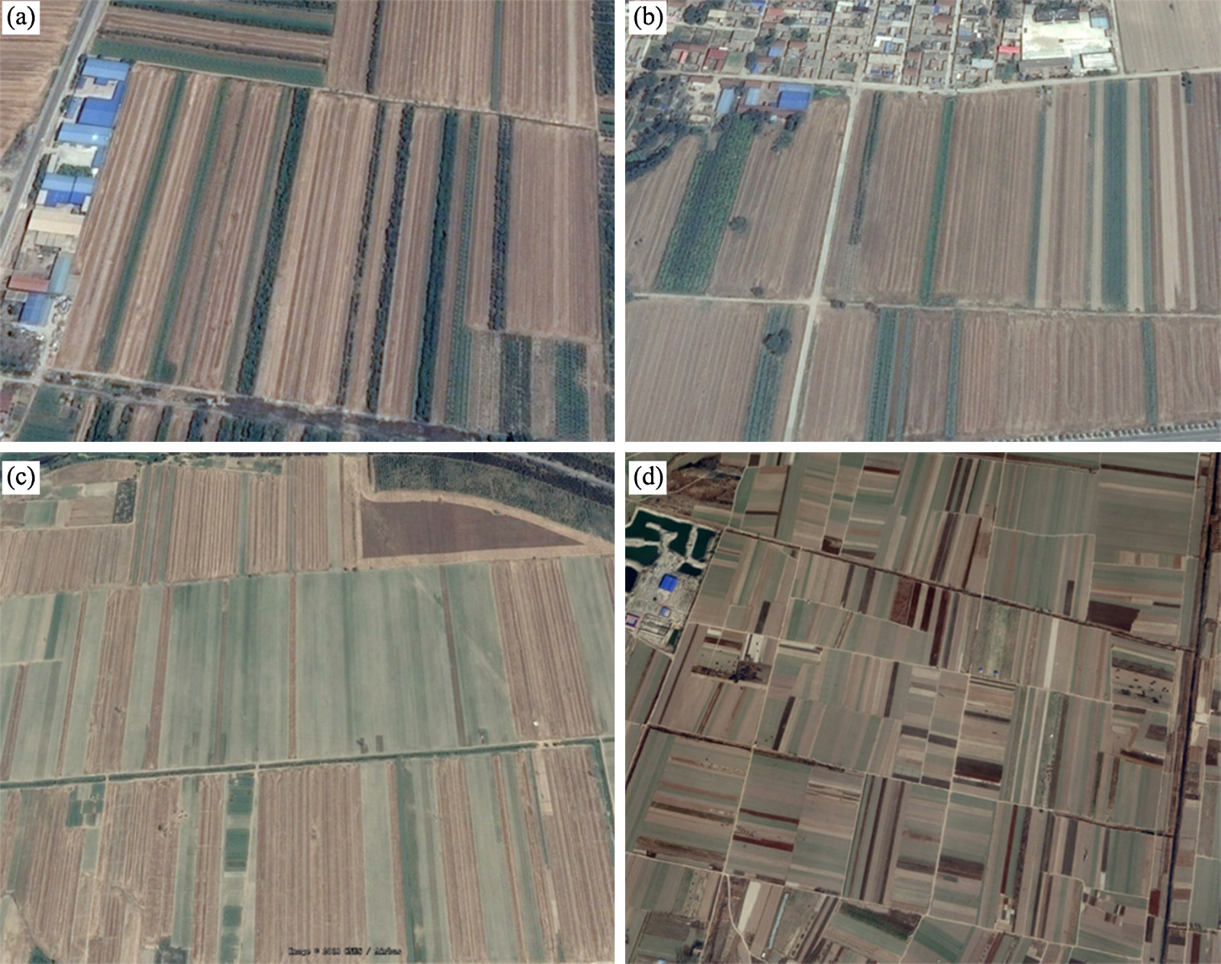
High-resolution images of the crop fields in the study area. (A) Sub-region 1, (B) Sub-region 2, (C) Sub-region 3, (D) Sub-region 4.

Hebei Province is located in the North China Plain and it is one of Chinese main cropping provinces. Hengshui City is situated between 115°10′–116°34′E and 37°03′–38°23′N, and occupies an area of 8.12 × 10^5^ ha, of which farmland occupies 5.71 × 10^5^ ha (approximately 70.3%) (Liu et al., 2015). The study region has a warm temperate continental monsoon climate that is characterized by hot, rainy summers and cold, dry winters. The main land cover/use types within this area are cropland, grassland, residential land, industrial land and water areas. The study area is representative of regions that rotate crops of winter-wheat and summer maize (Fig. 1). The major crop types in the study region are winter wheat, summer maize, spring maize and cotton. Winter wheat is harvested in June, while spring maize and cotton are mature in late August and September–October, respectively. Summer maize is sown after the harvest of winter wheat and is harvested in late September to early October. The croplands are mostly arranged in long, narrow fields (Fig. 2), which have a high potential for producing mixed pixels on Landsat images. In addition, shortages of water further affect crop production (Liu et al., 2015; Xie, Cheng & Lv, 2017). Therefore, early season crop type maps are urgently needed to improve crop growth monitoring and harvest management. Sentinel image may provide an opportunity to solve these problems.

The remote sensing data used in this study were Sentinel-1 and Sentinel-2 data obtained from April 1 to October 31, 2017. Sentinel-1A provides C-band images at a 6-day revisit frequency. The Level 1 ground range detected (GRD) product in the interferometric wide swath model was used in this study. The images have dual-polarization vertical transmission/vertical receiving (VV) and vertical transmission/horizontal receiving (VH) bands. The images have high geometric accuracy and were provided as backscatter coefficient in decibels. Sentinel-2 data were provided at 10 m spatial resolution with a 5-day revisit frequency; such data has shown potential to identify crop types (Belgiu & Csillik, 2018). There were 96 scenes of Sentinel-1 images and 254 scenes of Sentinel-2 images utilized in this study. All of the Sentinel-1 and Sentinel-2 data were processed using JavaScript application programming interfaces on the Google Earth Engine platform (GEE), which provides a fast and large-area computational service (Google, 2015; Gorelick et al., 2017). The spatial resolution of Sentinel-1A images were 10 m on the GEE, and the data is available in GEE as the “COPERNICUS/S1_GRD” image collection. A 15-day composition was used to process the Sentinel-1 data, and the maximum value among each 15-day composition period was used to represent the Sentinel-1 signal of the 15-day period. Of the 14 composited images, all were used in this study except for the July 1–15 image, because it was not fully covered by effective observations (Table 1). Sentinel-2 data were Level 1C top of atmosphere (TOA) reflectance data (image collection COPERNICUS/S2 on GEE). Cloud-cover pixels were selected and removed using the automatic phenology-based synthesis classification algorithm (Simonetti et al., 2015), and 15-day composition image time series were then generated on GEE, with the middle value of each composition period used in the composition. As the spatial resolution of the blue band (Band 2), green band (Band 3), red band (Band 4) and near-infrared (NIR) band (Band 8) were 10 m, we only included these four bands. Next, the normalized difference vegetation index (NDVI) for each composition period was calculated using the corresponding TOA reflectance (Rouse et al., 1974). Among the 14 composited images generated, four had cloud cover that was greater than 50% and were therefore discarded. The Sentinel-2 data used in this study is listed in Table 1.

**Table 1:** Crop calendars of the study area and satellite data acquired.

Composition periods	Winter wheat	Cotton	Spring maize	Summer maize	Sentinel-1	Sentinel-2
April 1st–15th	Developing				Y (8)	N
April 16–30th	Developing				Y (4)	Y (12)
May 1st–15th	Developing	Sowing	Sowing		Y (10)	N
May 16th–31st	Developing	Developing	Developing		Y (6)	Y (18)
June 1st–15th	Maturation	Developing	Developing		Y (8)	Y (17)
June 16–30th	Maturation	Developing	Developing		Y (6)	Y (15)
July 1st–15th		Developing	Developing	Sowing	N	Y (17)
July 16th–31st		Developing	Developing	Developing	Y (8)	N
August 1st–15th		Developing	Developing	Developing	Y (6)	Y (27)
August 16th–31st		Developing	Maturation	Developing	Y (10)	Y (12)
September 1st–15th		Maturation	Maturation	Developing	Y (6)	N
September 16–30th		Maturation		Maturation	Y (4)	Y (25)
October 1st–15th		Maturation		Maturation	Y (6)	Y (9)
October 16th–31st					Y (10)	Y (17)

**Notes:**

The “Y” indicates that the 15-day composition data was acquired in the corresponding composition period, and “N” indicates that the data was not acquired. The number in the table indicates the number of images used for the corresponding composition.

Crop fields are typically long and narrow, at only 20–50 m wide, so that the images with 20/30 m spatial resolution have a lot of mixed pixels representing multiple crops. Therefore, we only used the four optical bands (Band 2 blue, Band 3 green, Band 4 red, Band 8 NIR) of Sentinel-2 images and the NDVI calculated from the optical bands with 10 m resolution. In addition, we generated 15-day composite images from the Sentinel-1 and Sentinel-2 images, the 15-day composite images reduce the frequency of missing value pixels and described crop growth effectively. During the image composition procedure, we simply calculated TOA reflectance using the median value of all available observations in the composition period. We did not consider the solar zenith angle, observation angle and bidirectional reflectance distribution function, as the number of available observations was limited (Huete et al., 2002; Wardlow, Egbert & Kastens, 2007).

Ground reference data were collected during a field survey of Hengshui in August 2017. The surveyed fields were selected to include the major crop types across the study areas. A total of 373 fields were surveyed in-situ, and the crop types and geographic coordinates of the surveyed fields were recorded. Afterward, the boundaries of the surveyed crop fields were digitized as polygons using ArcGIS. For each crop type, the polygons were equally divided as training polygons and validation polygons. Next, the polygons were converted to Sentinel-2 pixels and boundary pixels were removed to ensure that all samples contained pure pixels. Finally, 17,542 training samples (pixels) and 25,124 validation samples (pixels) were acquired. The classifier was trained using the training samples and the classification results were validated using the validation samples. This study used all validation samples to verify the classification accuracy because the pixel-based classifier was employed in this study. The numbers of surveyed polygons and training/validation samples are shown in Table 2.

**Table 2:** Number of training and validation samples.

Crop type	Number of surveyed polygons for training	Number of samples for training	Number of surveyed polygons for validation	Number of samples for validation
Cotton	23	1,811	24	1,944
Spring-maize	12	946	15	576
Summer-maize	10	802	12	1,372
Wheat-maize	79	14,287	78	14,763
Orchard	17	2,523	21	2,462
Seedling nursery	13	854	14	1,011
Woods	27	4,412	28	2,996

**Notes:**

“Spring-maize” fields contained maize planted in April, while “Summer-maize” was planted in June. “Wheat-maize” denotes fields where winter-wheat and summer maize crops were rotated (which is the most popular crop rotation in the North China Plain).

## Methodology

### Methodology overview

**Figure 3:**
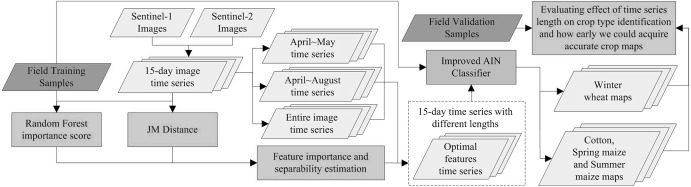
Flowchart illustrating the process of this study.

Figure 3 depicts the flowchart of this study. First, 15-day compositions of Sentinel-1 and Sentinel-2 data were generated. As winter wheat was harvested in June and cotton, spring maize and summer maize were harvested in September, we used images from April to May for early-season winter wheat identification and images from April to August for early-season cotton, spring maize and summer maize identification. We firstly used Jeffries–Matusita (JM) distance and the Gini importance score from the random forest (RF) algorithm to evaluate the crop separability and contribution of each feature on crop classification, and generated image time series of the optimal bands. Then, we proposed an improved artificial immune network (IAIN) which could deal with the missing values in the image time series. Finally, we varied time series length, used IAIN to identify crop types and evaluated the effect of time series length on crop classification accuracies. Based on these results, we determined how early the accurate distribution maps of winter wheat, cotton, spring maize and summer maize can be acquired.

### Feature importance estimation

We used both Gini importance scores from the RF algorithm and JM distances to select the optimal features for early-season crop type classification (Breiman, 2001; Bruzzone, Roli & Serpico, 1995). The JM distance ranges from 0 to 2, and larger JM distance indicates higher level of separability between the two classes. We selected JM distance in this study to estimate pair-wise crop separability for each feature because previous studies proved that JM distance have high potential to measure crop separability (Medjahed et al., 2016), and the JM distance was implemented in Matlab 2012B in this study. In addition, Gini index generated from RF was used to measure the importance of the features when considering all crops. The RF model combines multiple classification trees, and when training the RF model, each tree is constructed using two-thirds of the training records, the remaining records are used for a test classification with an error referred to the “out-of-bag error”. There are two free parameters of RF; the number of trees (ntree) and the number of features to split the nodes (mtry) were defined as 1,000 and the square root of the total number of input features in this study. During the training procedure of RF, every time a split of a node is made on a variable, the Gini impurity criterion for the descendent nodes is less than that of the parent node. The importance score is the sum of the Gini decreases for each individual variable over all trees in the classification forest. The Gini importance score were implemented using the Randomforest package in R (Breiman et al., 2013). Finally, the optimal features for identifying crop types in Hengshui were selected by considering both RF Gini index and JM distance.

### The improved artificial immune networks classifier

In this study, we IAIN algorithms for crop type identification. The IAIN methods are inspired by the human immune system and have shown high potential to solve pattern recognition problems (Gong, Im & Mountrakis, 2011). There are two procedures in IAIN: *training* and *classification*. The training procedure is to use training samples as “antigens,” then generate “antibodies” from the “antigens.” The classification procedure uses “antibodies” to classify images by identifying new crop “antigens.” Normally, “antibodies” contain three attributes: the crop type label of the antibody, the center vector, and recognition radius. An antibody can identify the antigens within its recognizing radius. “Antigens” contain two attributes: the crop type label and center vector.

After normalizing all features to a range of 0–1, the normal training procedures involve five steps: pre-selection, cloning, mutation, adaptive calculation of new antibodies and antibody reorganization (Zhong & Zhang, 2012). Pre-selection (Step 1) requires selection of an antigen which best represents the unrecognized antigens; Step 2 (cloning) is to clone a large number pre-selected antigens; Step 3 (mutation) is to mutate the cloned antigens randomly and generate “possible antibodies.” Afterward, the most complex step (Step 5, adaptively calculating new artificial antibodies) uses all “possible antibodies” to identify all the unrecognized antigens. The antibody that can identify the most antigens is then defined as a new antibody. Antigens that can be recognized by the new antibody are labeled as recognized antigens, and the other antigens remain as unrecognized antigens for generating the next antibody. Finally, when all antigens have been identified by the antibodies, the training procedure concludes. During the training procedure, a similarity measurement is used to estimate the distance between the antibody/antigen center vectors. This study used the Euclidean distance for this purpose as it is easily calculated (Lhermitte et al., 2011).

**Figure 4:**
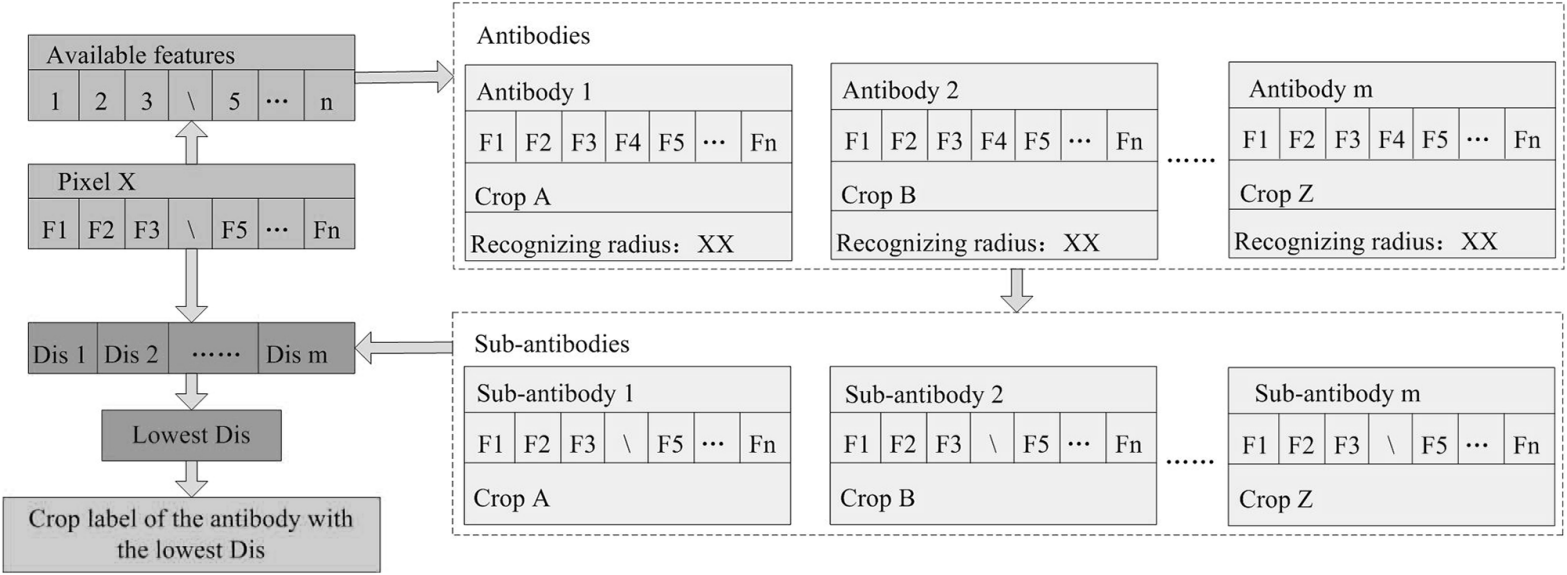
Classification procedure of the improved artificial immune networks (IAIN) algorithm. Note: This figure shows the condition that the feature F4 is left out.

To deal with image time series containing missing values, we IAIN classifier in the classification procedure by adaptively selecting classification features from “antibodies” (Fig. 4). For example, if the “antibodies” have *n* features but feature “F4” of pixel *x* is missing, the IAIN first detects the indexes of the available features of pixel *x*, then generates “sub-antibodies” only containing the available features of pixel *x*. Then the Euclidean distances between pixel *x* and the center vectors of all “sub-antibodies” are calculated. Finally, the pixels are labeled according to the crop type label of the antibody with the lowest distance.

### Early-season crop identification evaluation

To evaluate the earliest time phase that we could accurately identify the major crops in the study region, we set the April 16–30th as the start point, and extended the time series length by 15-day. The April 16–30th was set as the start point of this study because the Sentinel-2 data of April 1–15th composition contain too few cloud-free pixels. Next, we acquired several image series with different lengths, and then used the IAIN to classify these image time series. The classification accuracies were then assessed using the validation samples, and the influence of time series length on crop identification accuracies were evaluated based on these classification accuracies. Commonly, the classification accuracies increase with the time series length, and then become saturated. We compared the time phases that classification accuracies saturated and the harvest time of each crop, and finally deduced how long we could generate crop type distribution maps before the harvests.

### Non-vegetation masks

**Figure 5:**
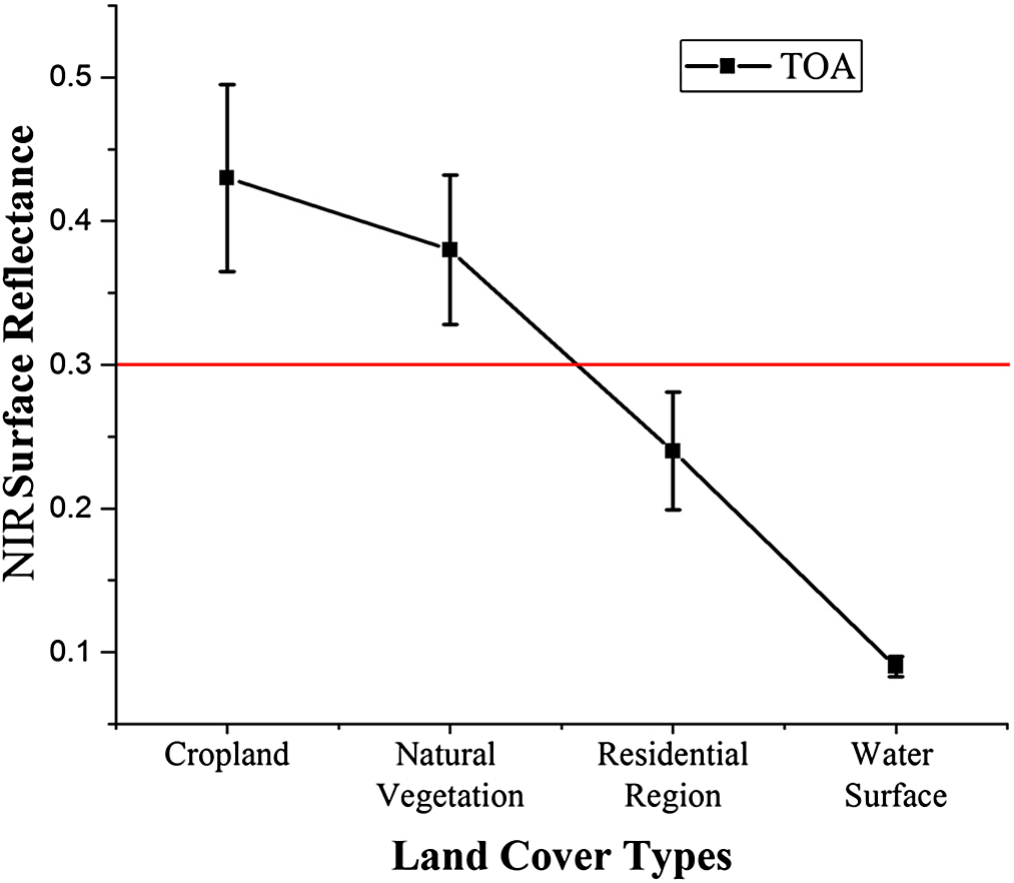
Threshold selection for the sparse vegetation mask mapping.

Although the time series signals of different crops have unique characteristics, there can be some noise caused by miscellaneous factors such as residential regions. We generated a sparse vegetation mask which could remove alkaline land, residential regions, water surfaces and other land with low vegetation cover. The mask used the maximum NIR TOA value during the growing season as the NIR band shows good potential for discriminating between vegetation and non-vegetation (Zhu & Woodcock, 2014). Figure 5 shows the maximum NIR TOA values of different land cover types during the growing season. The threshold was defined by subtracting the mean value of vegetation land cover (cropland and natural vegetation) from the standard deviation. In this study, the non-vegetation threshold was defined as 0.3.

## Results and Discussion

### Optimal feature selection

**Figure 6:**
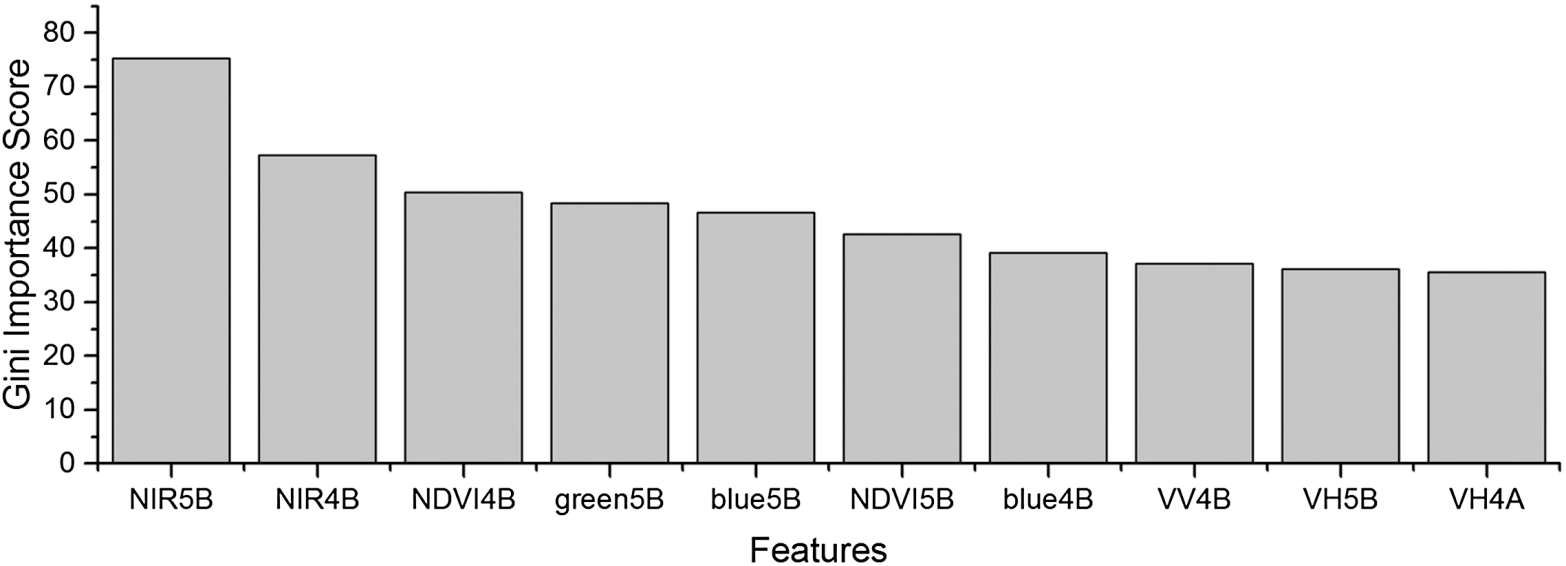
Top 10 features with high Gini importance scores for the April–May image time series. 4 = April, 5 = May, A = the composition for the 1–15th of each month, B = the composition for the 16–30th/31st of each month. For example, NIR5B = the NIR band of the May 16–31 composition.

**Figure 7:**
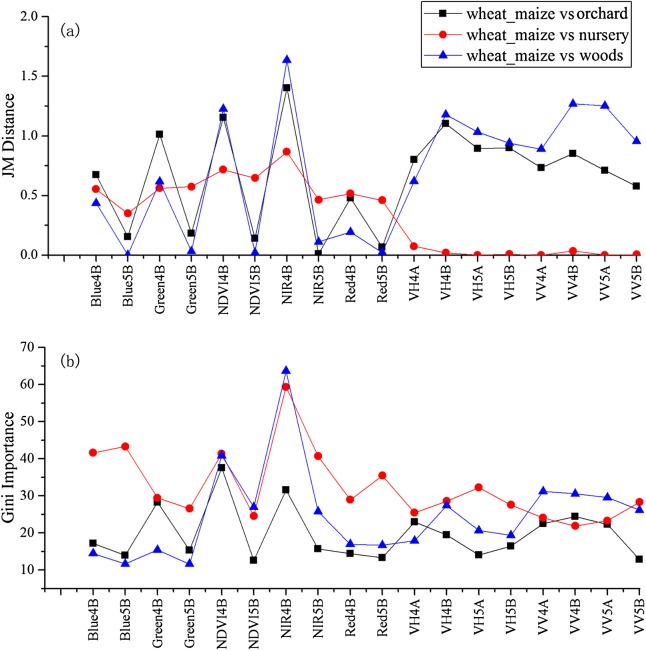
JM distance and Gini scores of the features for orchard, woods, seedling nursery and winter wheat. (A) JM distance; (B) Gini importance score.

**Figure 8:**
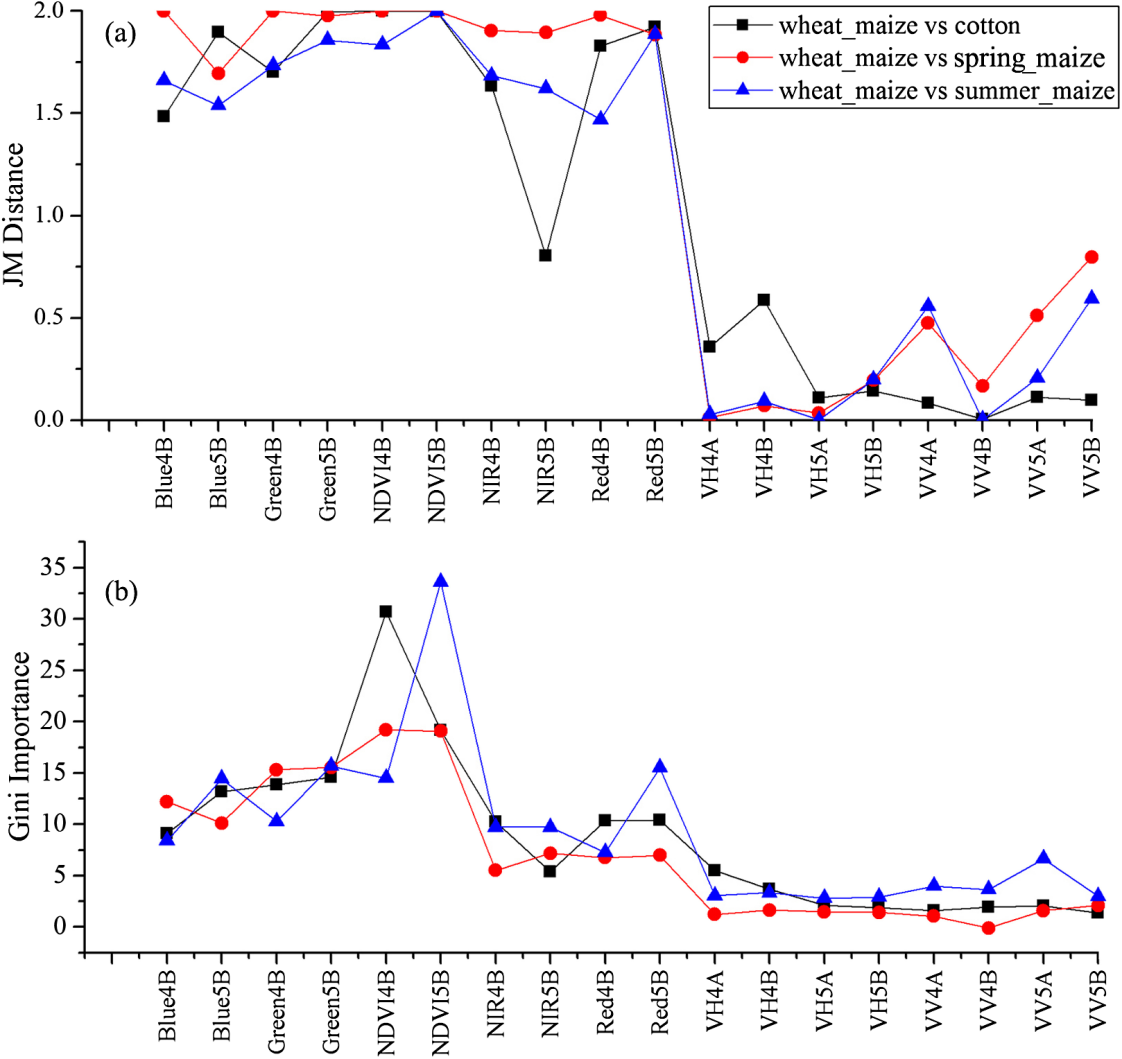
JM distance and Gini scores of the features for cotton, spring maize, summer maize and winter wheat. (A) JM distance; (B) Gini importance score.

As the winter wheat was harvested in early June, we used the April and May features for early-season winter wheat identification. When considering all crop types, the Gini score for the May NIR band was 75.3 (Fig. 6), which was the highest among all features during April and May. Then, pair-wise JM distances and Gini importances were calculated for each feature (Tables S1 and S2 in the supporting files). Figures 7 and 8 show that all four optical bands and the NDVI of April 16–30 and May 16–31 periods had high JM distances (generally higher than 1.5) and Gini scores (higher than 30) when separating winter wheat from cotton, spring maize and summer maize. Therefore, the optimal features and NDVI were used for winter wheat classification (Table 3). For the April 1–May 15 periods, we also included VH and VV data of May 1–15 as Sentinel-2 optical images were not acquired in this time phase.

**Table 3:** Features for early-season winter wheat identification with time series of different lengths.

Time series lengths	Gained features
April 1st–15th	VH and VV of April 1st–15th period
April 1st–30th	4 Optical bands and NDVI of April 16–30th
April 1st–May 15th	VH and VV of May 1st–15th period
April 1st–May 31st	4 Optical bands and NDVI of May 16th–31st

**Note:**

The features listed in this table were gained by increasing the time series duration. For example, for the time series increased from April 1–15 to April 1–30, 4 optical bands and the NDVI of April 16– 30 were added for early-season winter wheat classification.

**Figure 9:**
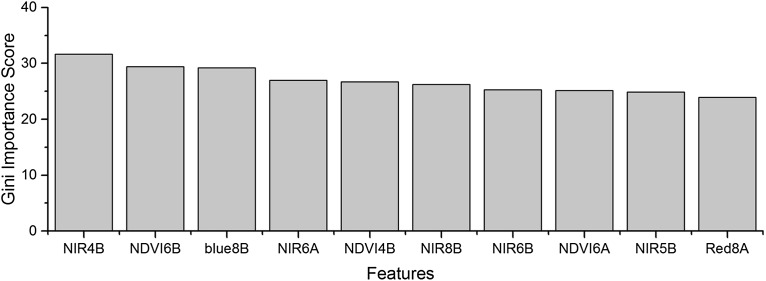
Top 10 features with high Gini importance scores for the April–August image time series. 4 = April, 5 = May, A = the composition for the 1–15th of each month, B = the composition for the 16–30th/31st of each month. For example, NIR4B = the NIR band of the April 16–31 composition.

**Figure 10:**
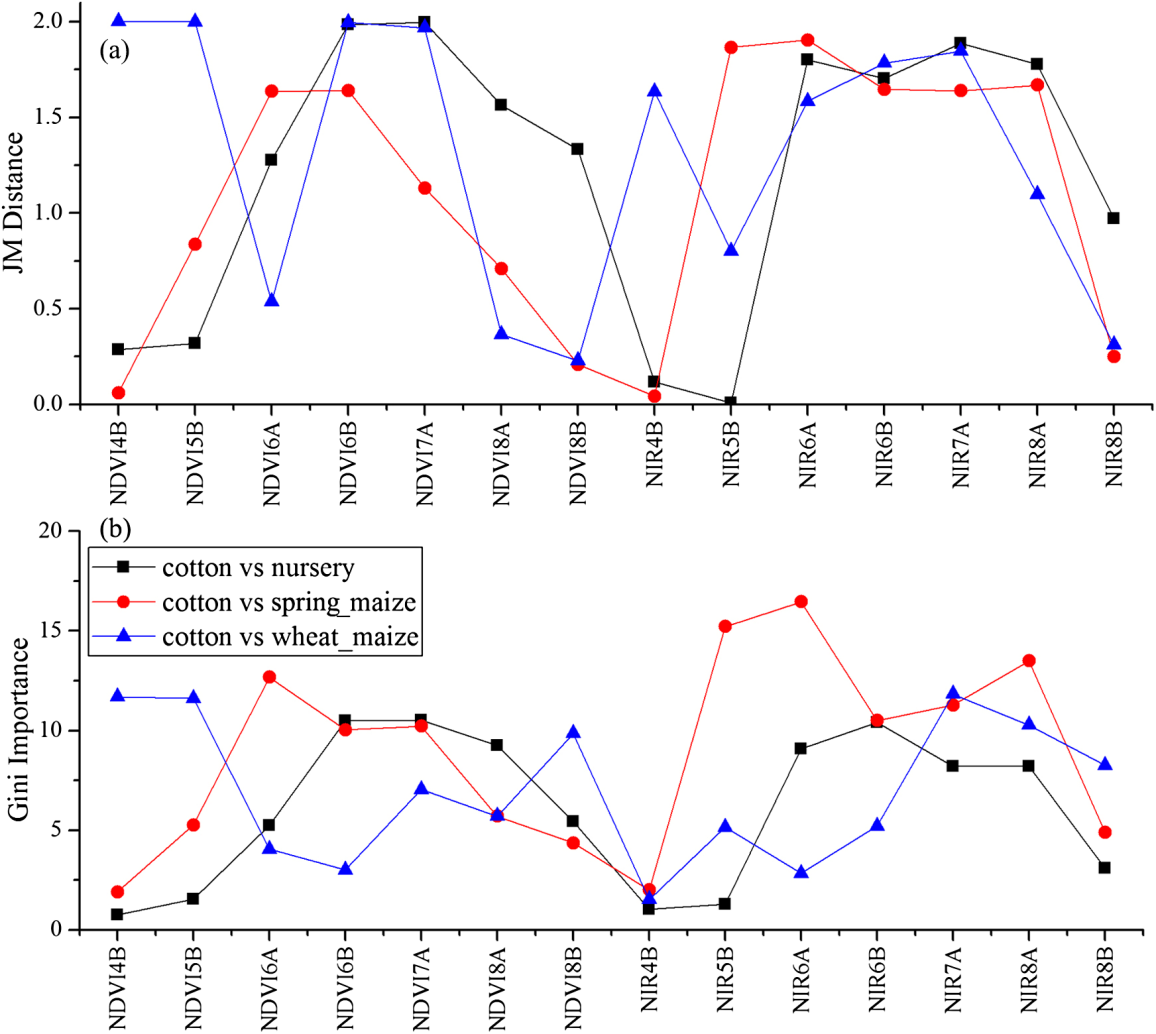
JM distance and Gini score of the features of seedling nurseries, spring maize, summer maize and cotton. (A) JM distance; (B) Gini importance score.

**Figure 11:**
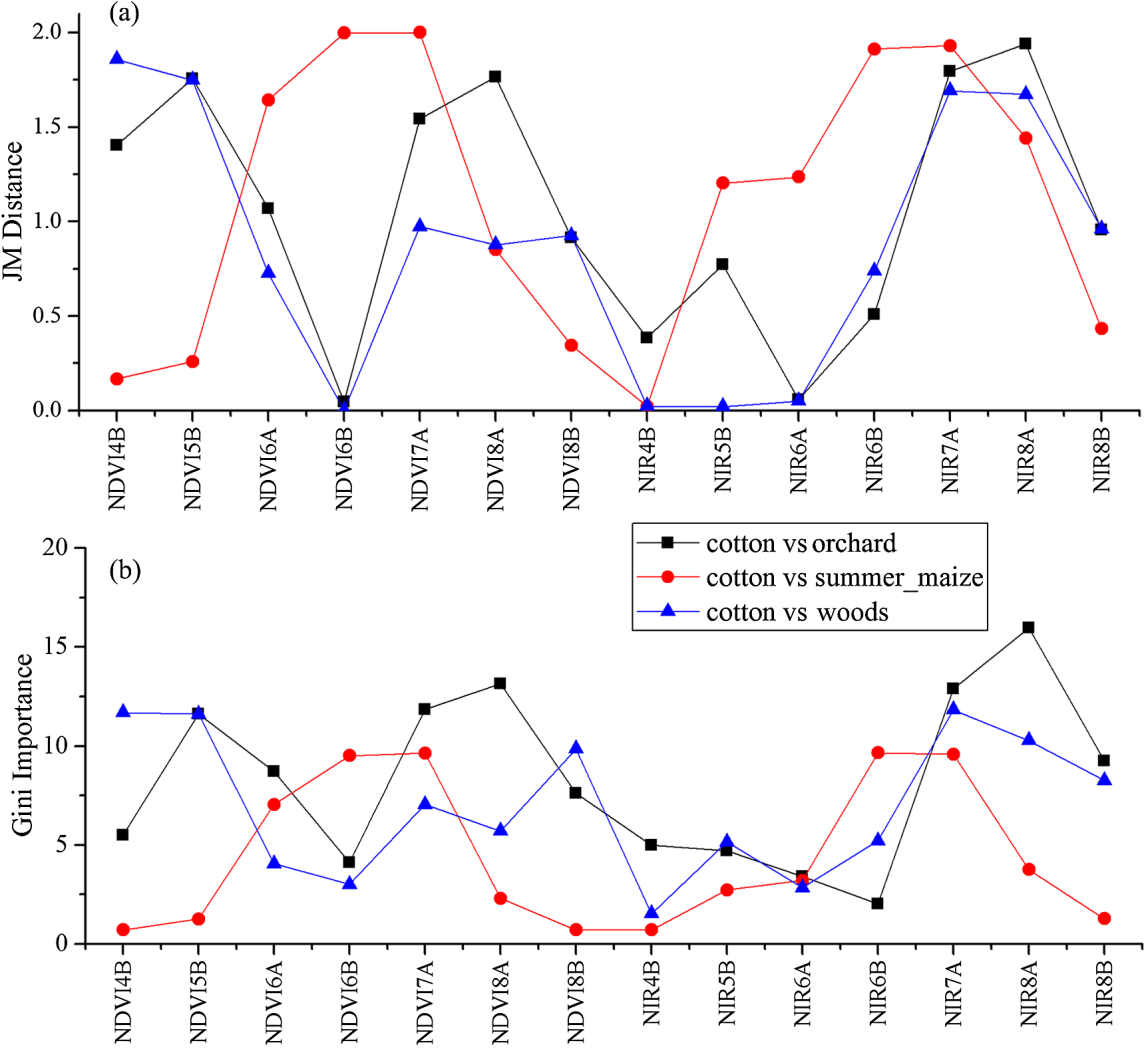
JM distance and Gini scores of the features of orchards, woods, summer maize and cotton. (A) JM distance; (B) Gini importance score.

**Figure 12:**
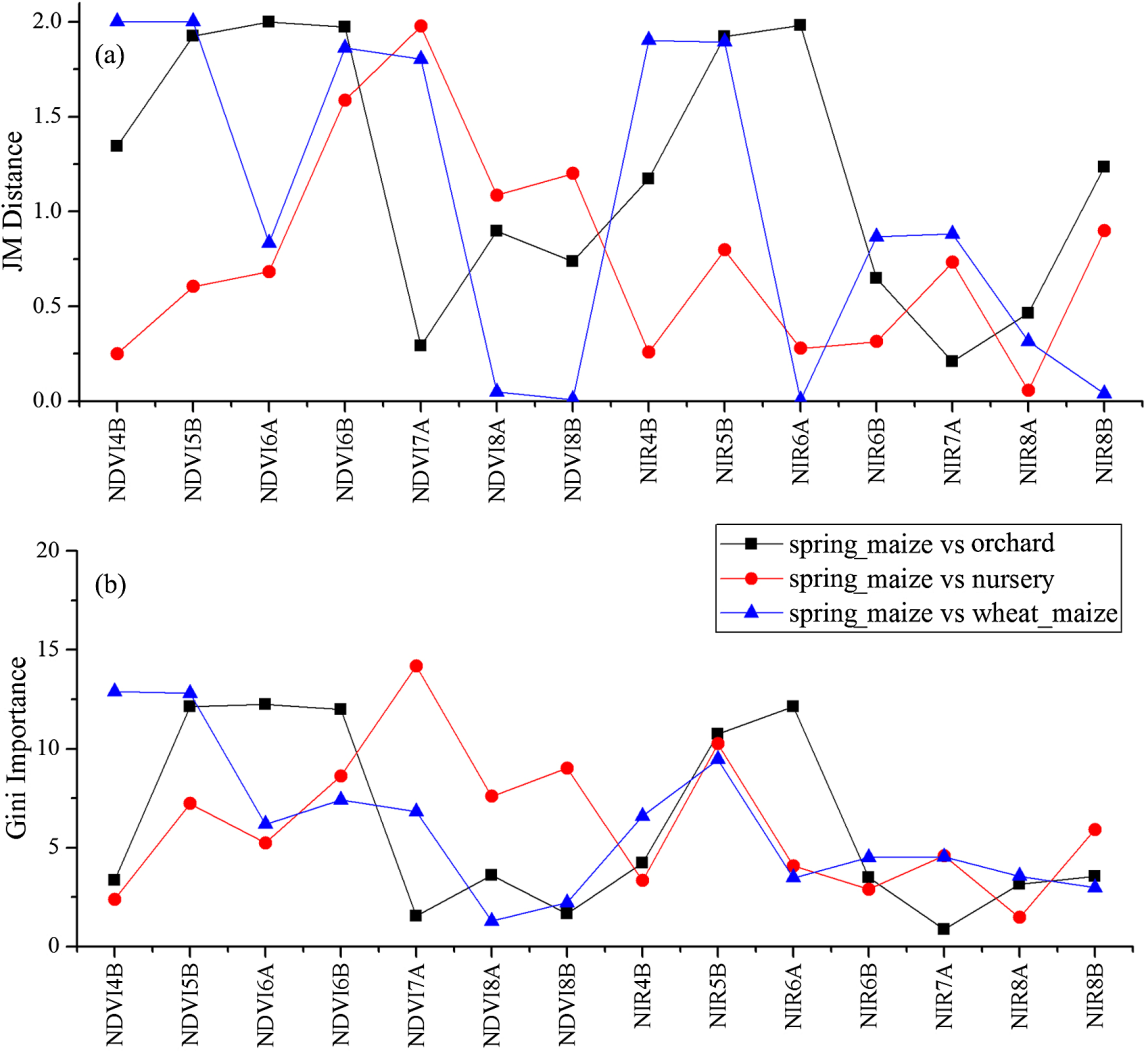
JM distances and Gini scores of the features distinguishing orchards, seed nurseries, wheat-maize and spring maize. (A) JM distance; (B) Gini importance score.

**Figure 13:**
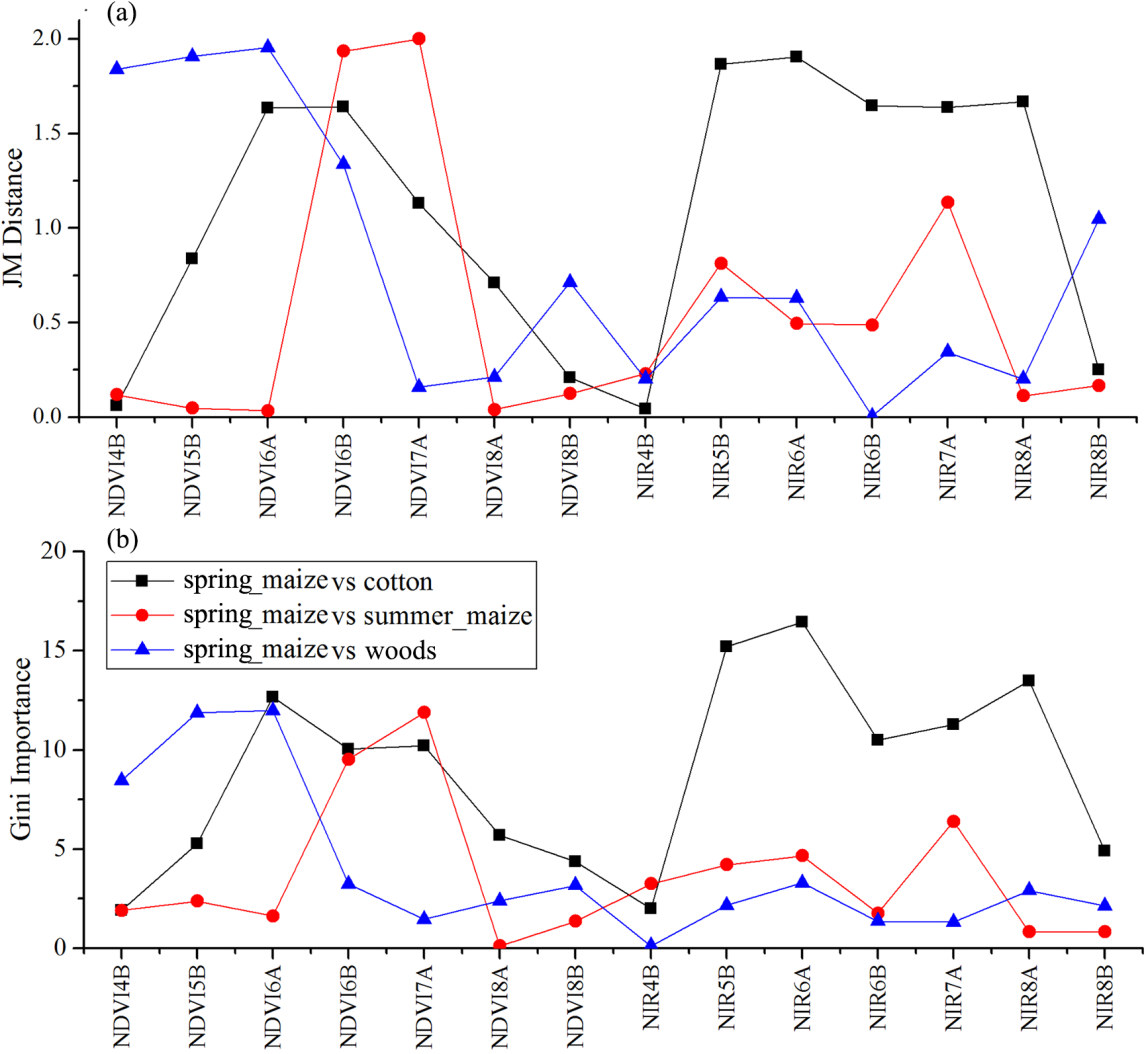
JM distance and Gini scores of the features distinguishing cotton, summer maize, woods and spring maize. (A) JM distance; (B) Gini importance score.

**Figure 14:**
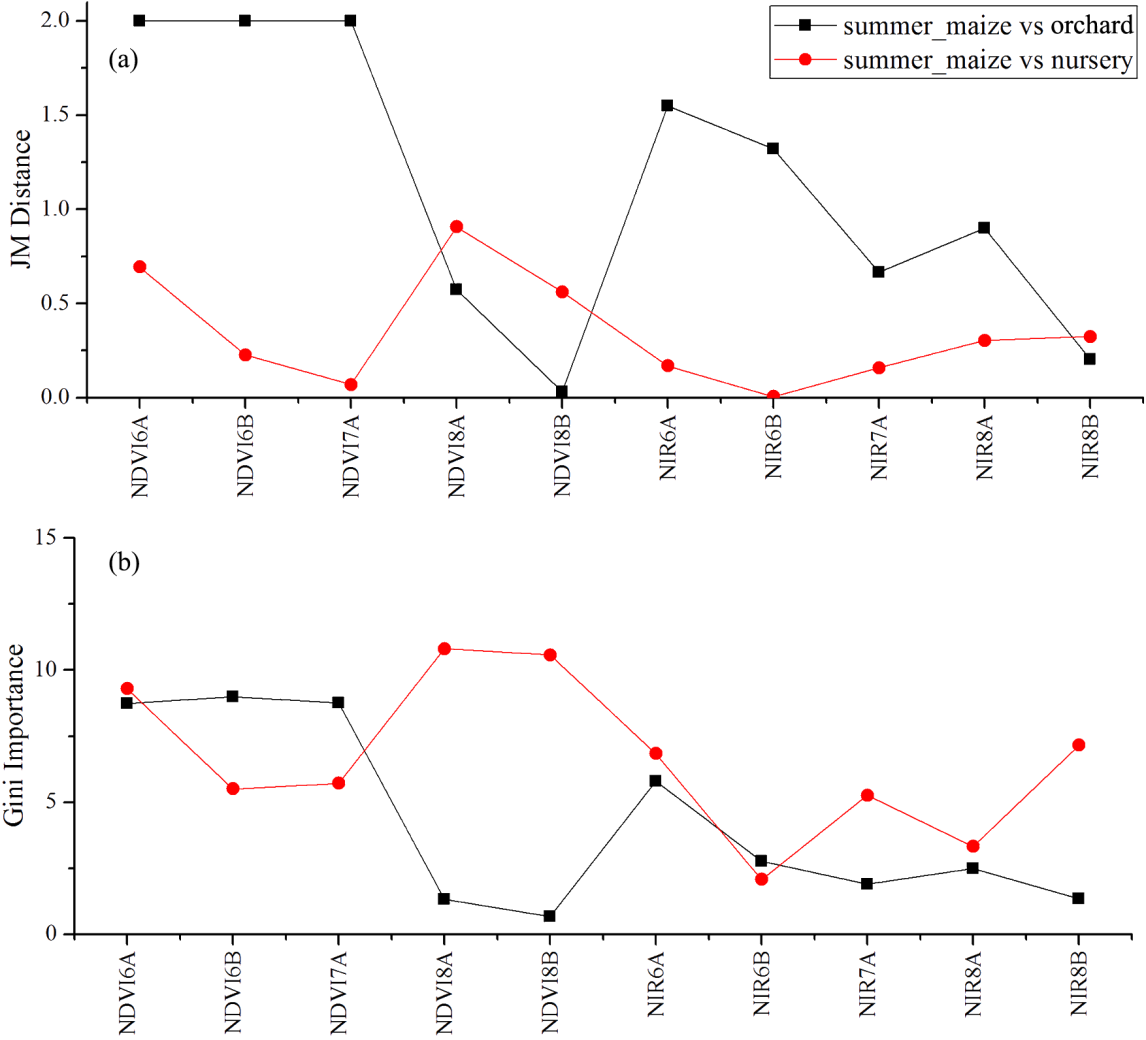
JM distance and Gini scores of the features distinguishing orchards, seeding nurseries and summer maize. (A) JM distance; (B) Gini importance score.

**Figure 15:**
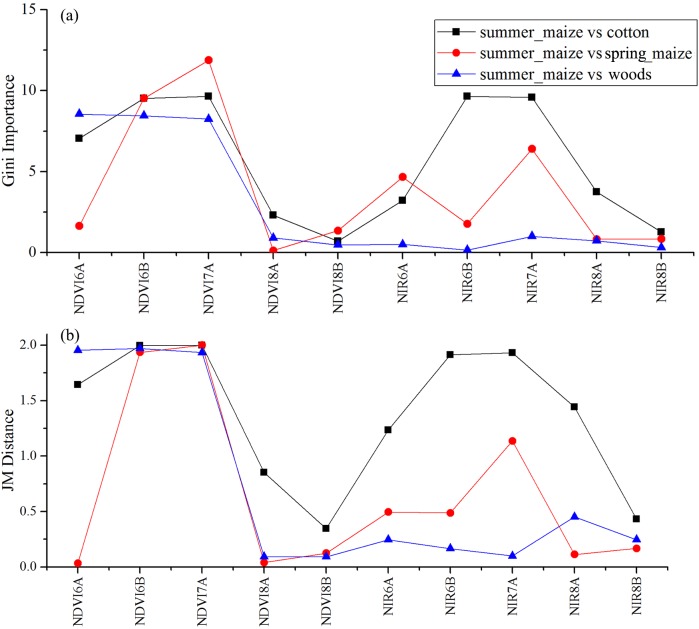
JM distance and Gini scores of the features distinguishing cotton, spring maize, woods and summer maize. (A) Gini importance score; (B) JM distance.

We used the image time series of April–August for early identification of the summer crops (cotton, spring maize and summer maize). Five NIR features (from the April 16–30, May 16–31, June 1–15, June 16–30 and August 16–31 compositions) and three NDVI features (April 16–30, June 1–15, June 16–30 compositions) were selected among the top 10 features with high Gini scores (Fig. 9). Figures 10 and 11 show that NDVI and NIR data of April and May could separate cotton from woods, orchards and winter wheat, as these crops green up earlier than cotton. Then, spring maize was separable from cotton using June features, and summer maize was separable from cotton using June and July 1–15 composition features. Figures 12 and 13 show that woods, orchards and winter wheat were separable from spring maize in April and May, and features of the June 16–30 and July 1–15 compositions could discriminate spring maize and summer maize. For summer maize identification, as summer maize is mainly sown in late June and early July, its feature separability and importance during June–August were estimated. Woods, orchards and cotton crops were separable from summer maize in June (Figs. 14 and 15). However, summer maize and seedling nurseries had lower separability as their JM distances were lower than 1.0 during June and August. Only the NDVI of the August 1–15 composition had relatively high JM distance (0.91). Generally, as NDVI and NIR contributed the most to separating the summer crops, we used NDVI and NIR time series identify summer crops in early season, VH and VV data of May 1–15 were included because Sentinel-2 data were not acquired (Table 4).

**Table 4:** Features for early-season summer crop identification with different time series lengths.

Time series lengths	Gained features
April 16–30th	NIR band and NDVI of April 16–30th
April 16th–May15th	VH and VV of May 1st–15th period
April 16th–May31st	NIR band and NDVI of May 16th–31st
April 16th–June 15th	NIR band and NDVI of June 1st–15th
April 16th–June 30th	NIR band and NDVI of June 16–30th
April 16th–July 15th	NIR band and NDVI of July 1st–15th
April 16th–July 31st	VH and VV of July 16th–31st period
April 16th–August 15th	NIR band and NDVI of August 1st–15th
April 16th–August 31st	NIR band and NDVI of August 16th–31st

**Note:**

Early season identification of spring maize and cotton were conducted using NDVI and NIR data from the April 16–August 31 time series, and the early-season summer maize was identified using NDVI and NIR data from the June 1– August 31 time series.

Feature contribution results showed that features from April and May could discriminate summer crops (such as cotton and spring maize) from natural vegetation (such as woods) in this study, as natural vegetation greens up earlier than crops. June was the fast growing season of cotton and spring maize; therefore, features from this period can be used to discriminate between them as they green up at different speeds (Wardlow, Egbert & Kastens, 2007). Among the features used in this study, NDVI and NIR had the highest separability and contributed most to crop classification. This is consistent with Zhu & Woodcock (2014), who observed that NIR could best discriminate between agricultural land and forest. Although texture features are effective for discriminating between cropland and woods in very high-resolution images, such as Gaofen-1 data at 2 m resolution (Turker & Ozdarici, 2011), these features were not considered in this study because the spatial resolution of the Sentinel images used was 10 m. Hao, Wang & Niu (2015a) reported that cotton and spring maize in Xinjiang were often confused, but this was not the case in this study. Basically, the optimal time phases selected for early season crop type classification varied in different years and study regions, as the growing season of crops have inter-annual and spatial phenological variation (Hao et al., 2016; Zhang, Tan & Yu, 2014). Therefore, when training to identify crop types at early season in a new study region, the contribution of the time phases for crop classification should be reevaluated.

### Classification accuracies of IAIN for “missing value” pixels

Although the 15-day composition method was used to improve the quality of the image time series and reduce the number of missing values, there are still some pixels containing “NaN” values. Meanwhile, most existing classifiers, such as maximum likelihood classification, support vector machine and RF, cannot deal with missing values, if the input data contain “NaN” value, the these classifiers will return “not classified” as output. In this study, we proposed IAIN classifier to deal with the “missing value” problem.

To assess the potential of the IAIN of dealing with “missing value” pixels, we firstly used April–June and April–August image time series to identify winter wheat and summer crops, and used validation samples with “missing value” to verify the classification accuracies. We acquired 4,293 samples during April–June for winter wheat validation, and 8,065 samples during April–August for summer crops validation. Results (Tables 5 and 6) show that IAIN could identify pixels with “missing value,” the OAs of winter wheat and summer crops were 99% and 98.55%, respectively. Only a few winter wheat, cotton and summer maize samples were mislabeled as “Non-crop.” While, as the number of spring maize was small (only 101 with “missing value”), the producer’s accuracy (PA) and user’s accuracy (UA) of spring maize classification maybe not 100% in reality.

**Table 5:** Confusion matrix of winter wheat identification using irregular time series and IAIN classifier.

	Winter-wheat	Non-winter	PA	UA
Winter-wheat	1,727	0	97.57	100
Non-winter	43	2,523	100	98.32

**Note:**

OA = 99.00% and Kappa coefficient = 0.9793.

**Table 6:** Confusion matrix of summer crops identification using irregular time series and IAIN classifier.

	Cotton	Spring maize	Summer maize	Non-crop	PA	UA
Cotton	1,045	0	0	0	98.21	100
Spring maize	0	101	0	0	100	100
Summer maize	0	0	4,348	0	97.8	100
Non-crop	19	0	98	2,454	100	95.45

**Note:**

OA = 98.55% and Kappa coefficient = 0.9754.

**Figure 16:**
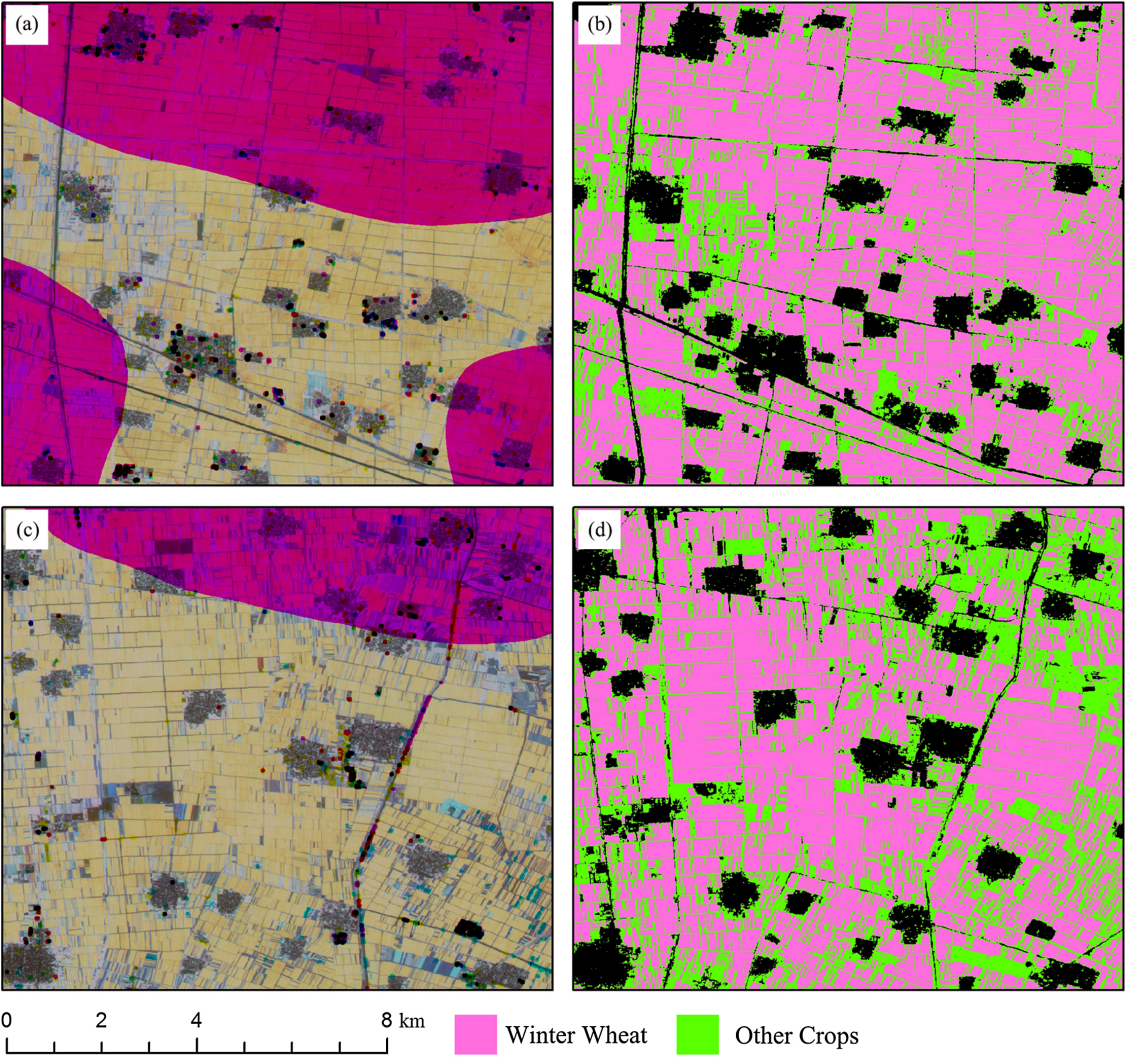
Subset images of winter wheat mapping for “missing value” patterns. (A) NDVI composed image (R: NDVI of April, G: NDVI of May, B: NDVI of June) of Sub-region 1; (B) Crop classification maps of Sub-region 1; (C) NDVI composed image (R: NDVI of April, G: NDVI of May, B: NDVI of June) of Sub-region 2; (D) Crop classification maps of Sub-region 2.

**Figure 17:**
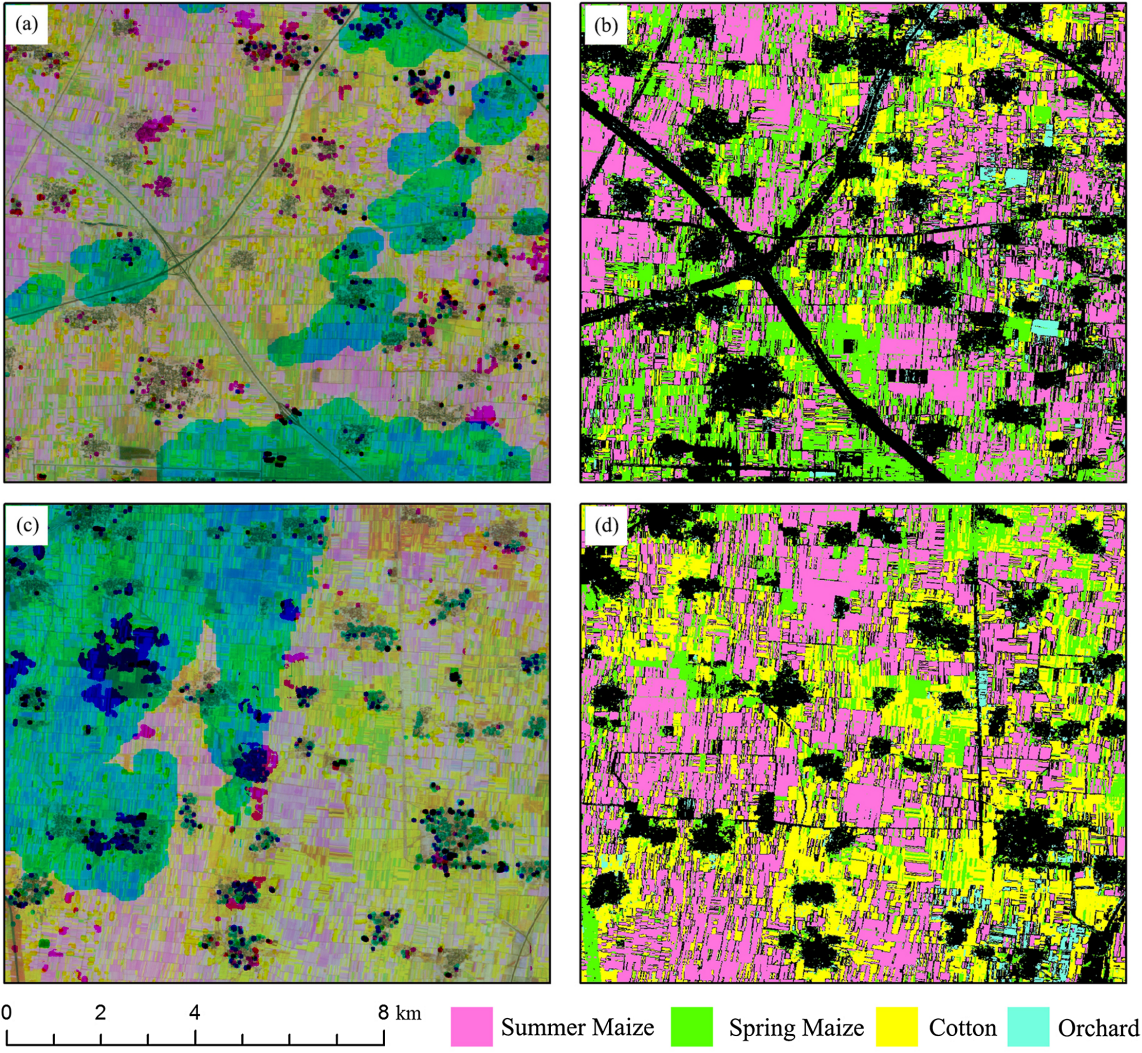
Subset images of summer crops mapping for “missing value” patterns. (A) NDVI composed image (R: NDVI of August, G: NDVI of June, B: NDVI of May) of Sub-region 1; (B) Crop classification maps of Sub-region 1; (C) NDVI composed image (R: NDVI of August, G: NDVI of June, B: NDVI of May) of Sub-region 2; (D) Crop classification maps of Sub-region 2.

Some subset images of winter wheat and summer crop classification maps are shown in Figs. 16 and 17. The red patterns in Fig. 16 and green/blue pattern in Fig. 17 were “missing value” patterns. And the classification results showed that the “missing value” patterns were generally correctly classified. However, there are still possible shortages when using IAIN classifier to deal with missing value. Firstly, the high separable features should be acquired for crop type classification, crops cannot be identified if only low separable features were used as inputs for the IAIN classifier. In addition, the similarity measurement used in this study is Euclidean distance, it is sensitive to the noise in the time series although it is easy calculating (Lhermitte et al., 2011). Thus, other similarity measurements could be considered to further improve the robustness of the IAIN classifier.

### Early-season winter wheat, cotton, spring maize and summer maize mapping

**Figure 18:**
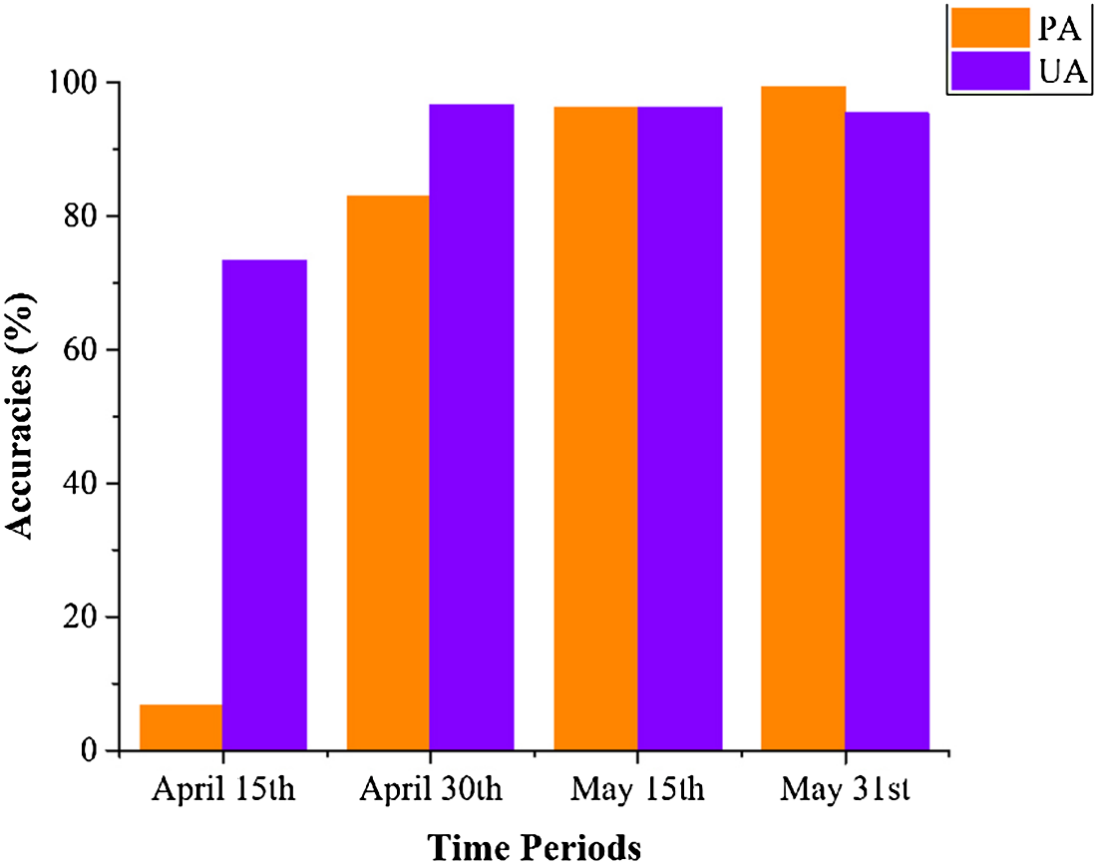
Producer’s (PA) and user’s accuracies (UA) of winter wheat with different time series lengths. April 15th = classification accuracies acquired using the April 1–15 image time series, April 30th = classification acquired using the April 1–30 image time series, and so forth for the other time series lengths.

**Figure 19:**
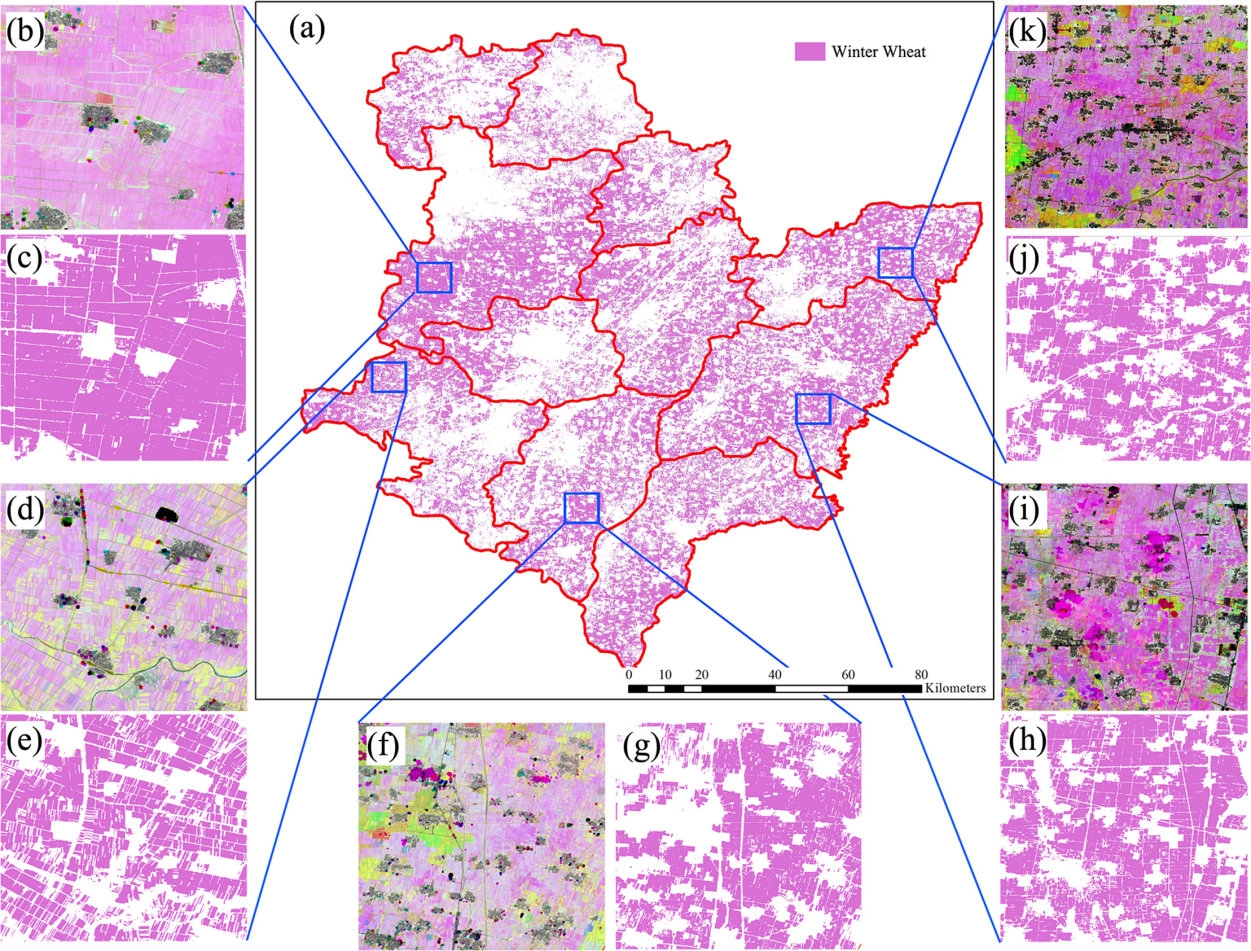
Spatial distribution of winter wheat in the study region (map acquired using the image time series of April 1–May 31, 2017). Sub-figure (A) Spatial distribution of winter wheat, (B, D, F, H, J) remote-sensed NDVI images composited by R: NDVI of September, G: NDVI of July and B: NDVI of May. As the winter wheat fields are rotated with summer maize, these fields have high NDVIs in September and low NDVIs in July, so that these were “pink parcels” on the composited NDVI image. (C, E, G, I, K) Winter wheat maps derived from the corresponding NDVI composited images.

Figure 18 shows that the VH and VV polarization data from the April 1–15 composition could not identify winter wheat, as both the PA and UA for winter wheat were low. Both PA and UA increased when the optical-band and NDVI data from the April 16–30 composition were used. For the April 1–May 15 and April 1–May 31 data, both PA and UA were higher than 95% (96.12% and 96.19% for May 15, and 99.28% and 95.42% for May 31, respectively). This indicated that winter wheat could be identified at May 15, which is approximately 20 days before the winter wheat harvest (early June). As both winter wheat and woody plants had high vegetation fractions, the misclassification of winter wheat is mainly due to confusion with orchards. This is consistent with the low separability of orchards, woods, seedling nurseries and winter wheat crops (Fig. 6). Figure 19 shows the spatial distribution of winter wheat generated from the April 1–May 31 data. Winter wheat was the dominant crop type in the study area and was present in each county. The winter wheat map was generated at 10 m resolution and shows the field boundaries clearly.

**Figure 20:**
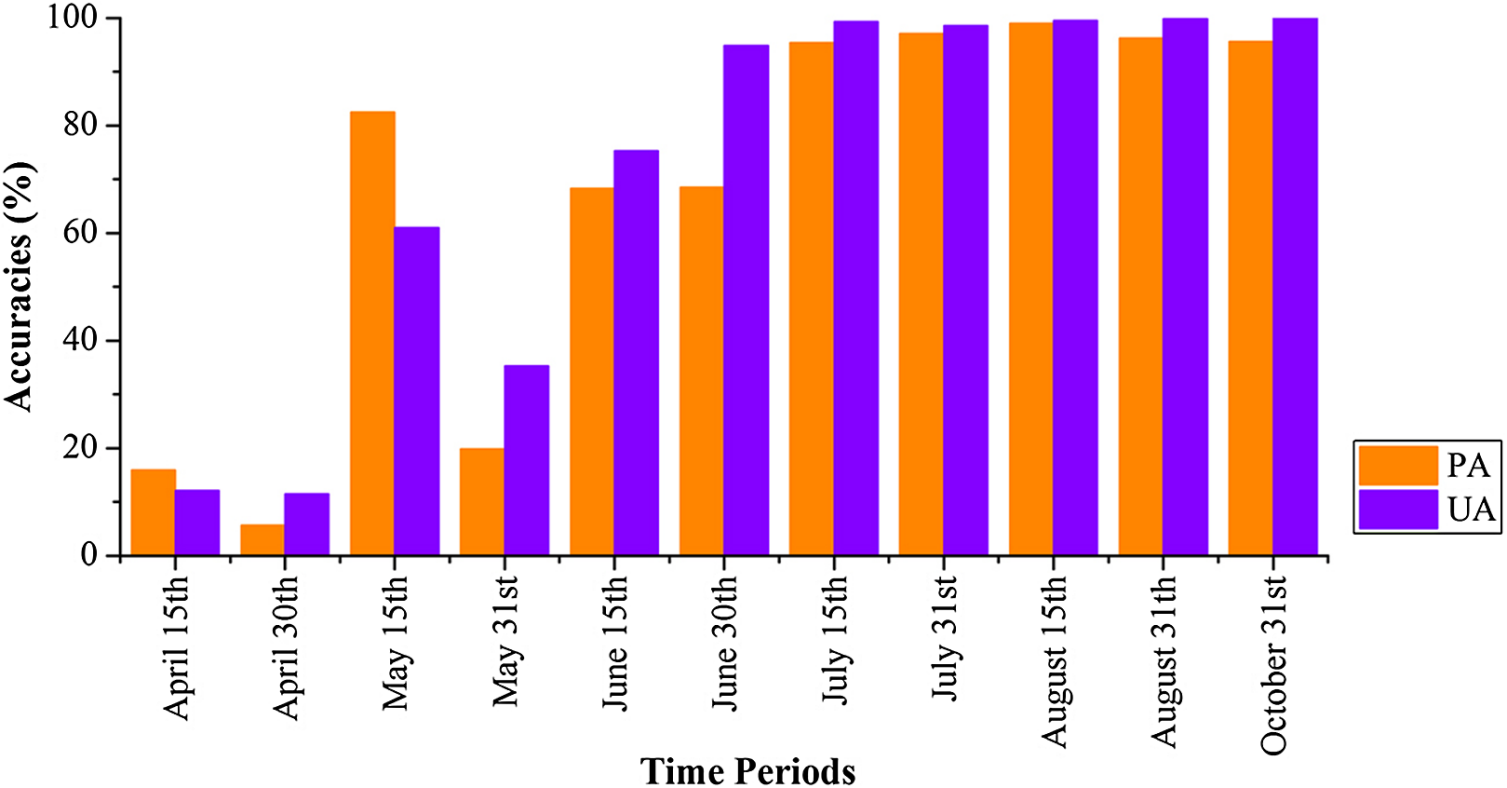
Producer’s (PA) and user’s accuracies (UA) of cotton with different time series lengths.

**Figure 21:**
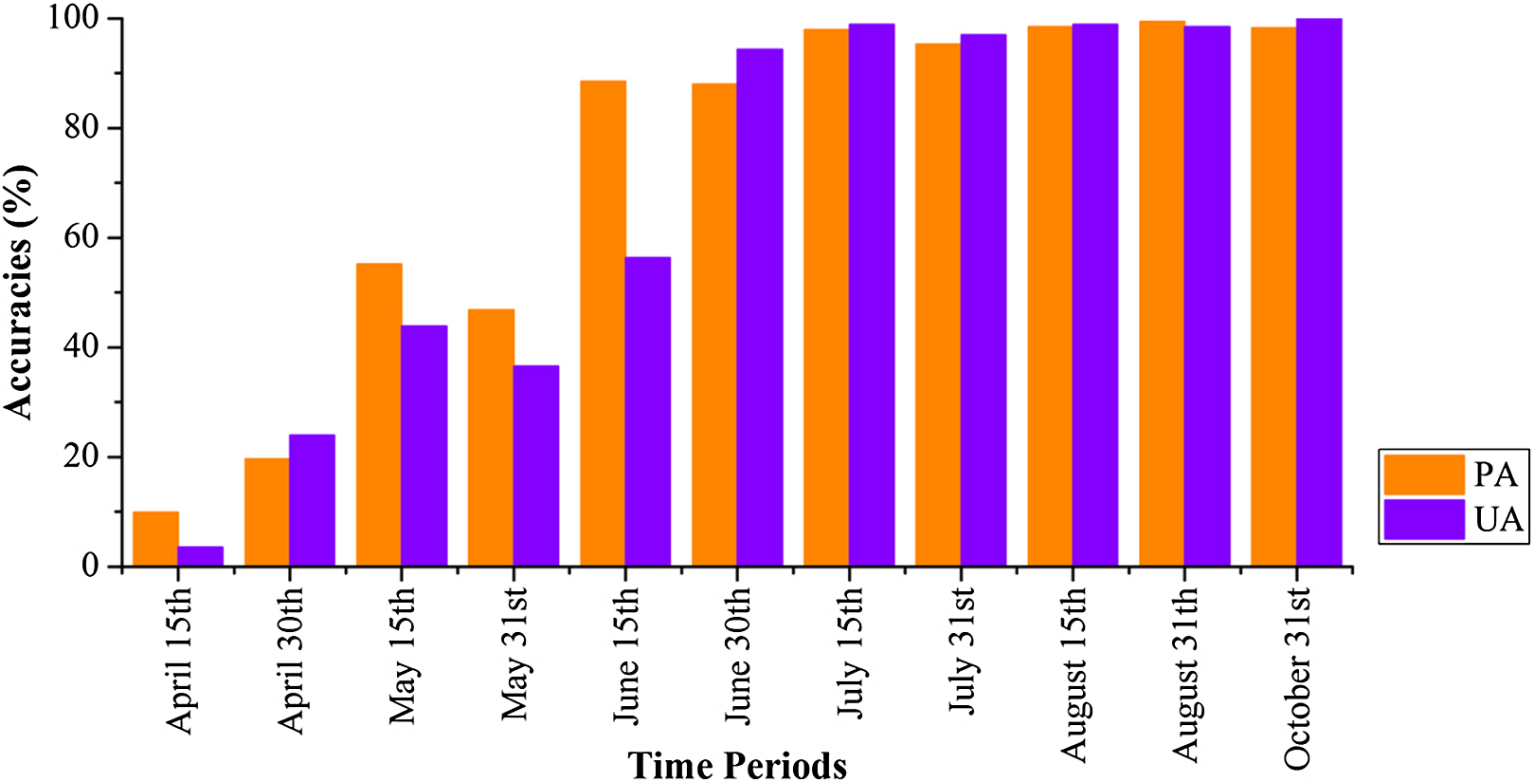
Producer’s (PA) and user’s accuracies (UA) of spring maize with different time series lengths.

Figures 20 and 21 show that images obtained during April 1–May 30 could not identify cotton and spring maize, as the PAs and UAs were lower than 60% in most cases. This was mainly because the crops were in their early stage, and the early signatures captured by satellites cannot discriminate between cotton and spring maize. When the June 1–15 image composition was used, the PAs and UAs of cotton and spring maize began to increase, which was consistent with the high JM distance between the two crops. Then, both the PAs and UAs of cotton and spring maize became greater than 95% when the length of the time series was increased to April 1–July 15 (PA and UA were 95.42% and 99.30% for cotton, and 97.92% and 98.95% for spring maize, respectively, at July 15). Use of the longer image time series did not increase the classification accuracies of these two crops. Spring maize was harvested in late August and cotton was harvested during September and October. Hence, an accurate distribution map was generated from April 1–July 15 data, which was more than one month before the harvests of these crops.

**Figure 22:**
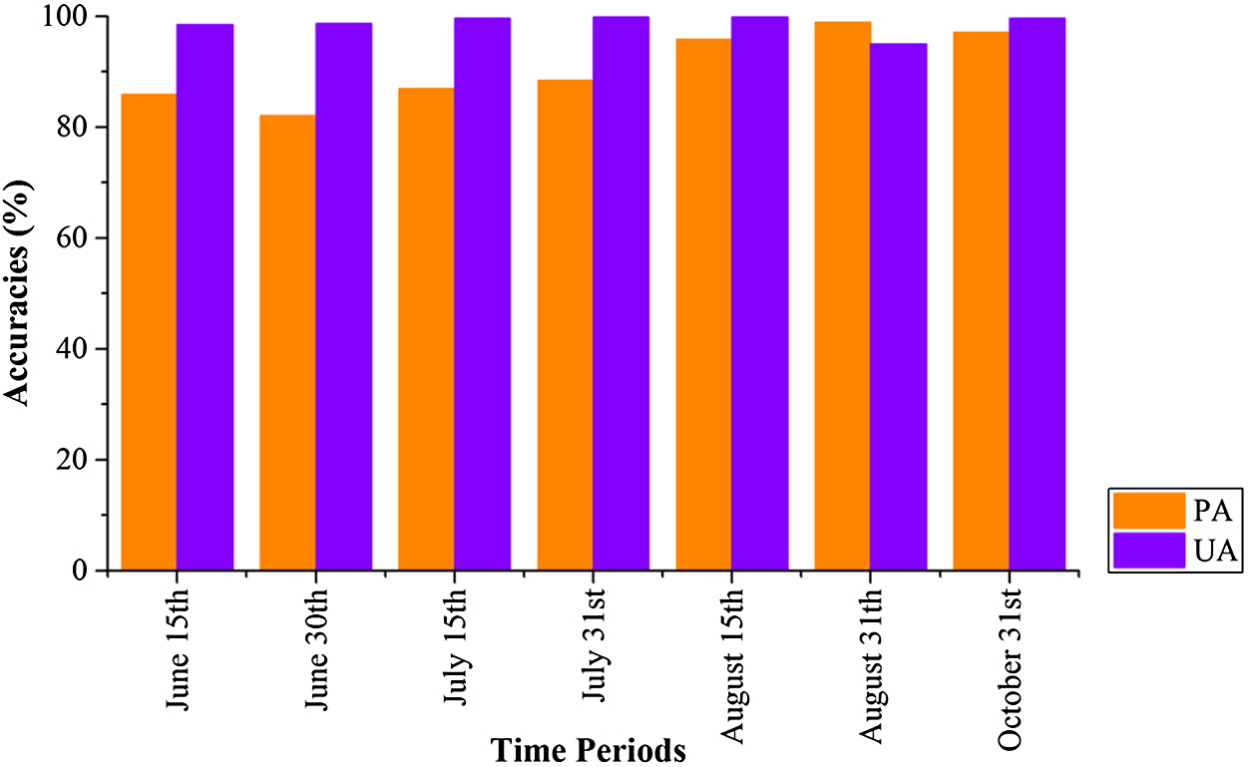
Producer’s (PA) and user’s Accuracies (UA) of summer maize with different time series lengths.

Figure 22 shows that PAs and UAs were higher than 80% using images composited from June 1–15 and June 16–30 data. The relatively high accuracies obtained for early-stage summer maize was consistent with its high separability (Figs. 15 and 20) caused by its low vegetation fraction in the early season. With image time series lengths of June 1–August 15, the PA and UA were higher than 95% (95.81% and 99.88%, respectively, for summer maize at August 15), and use of a longer image time series did not improve the classification accuracy. The summer maize was harvested in late September and early October. Hence, an accurate early summer maize map could be generated from August 15 data—one month earlier than the harvest.

**Figure 23:**
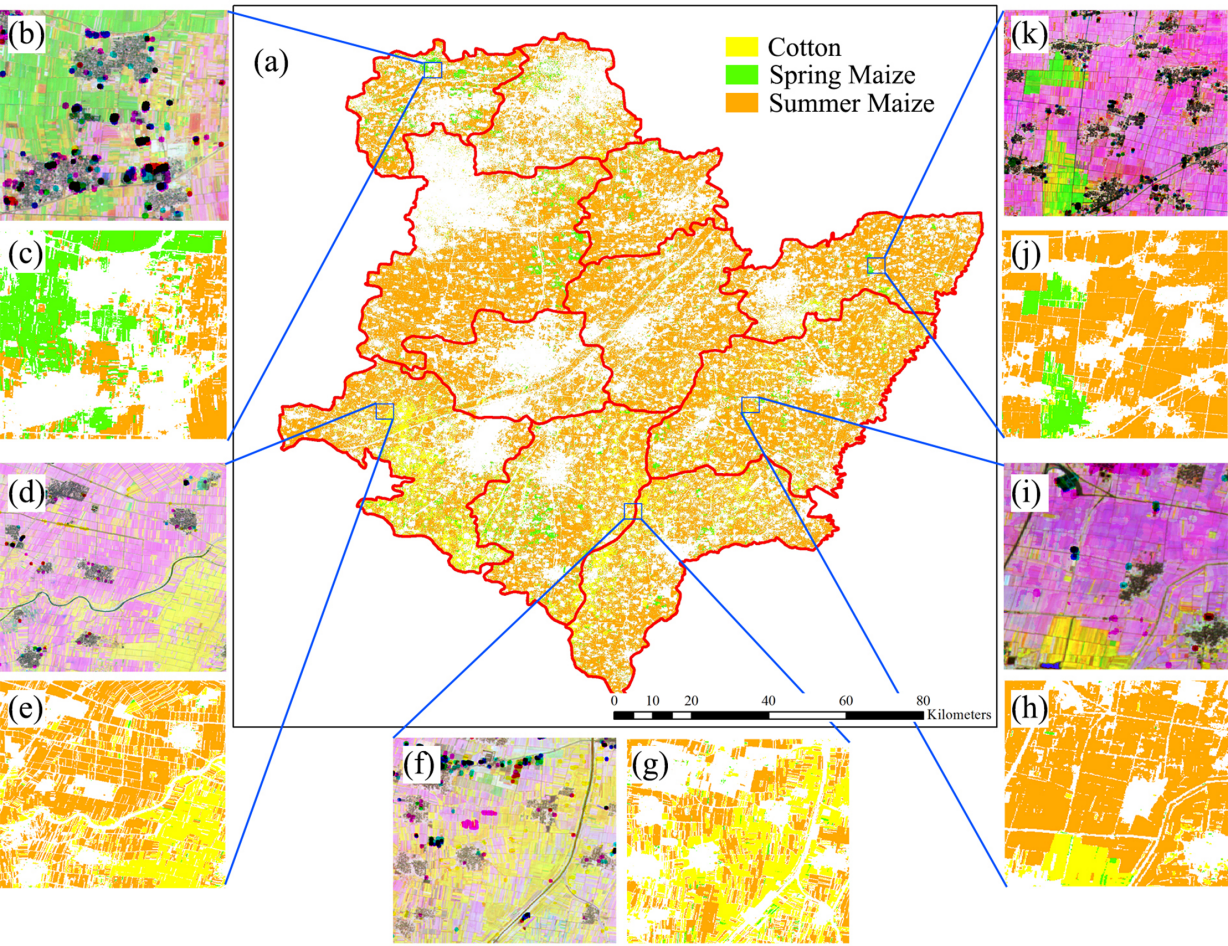
Spatial distribution of cotton, spring maize and summer maize in the study region (map derived from the April 1–August 15, 2017 time series). Sub-figure (A) is the spatial distribution of the winter wheat, the remote sensed images (sub-figures B, D, F, H, J) are NDVI images composited by R: NDVI of September, G: NDVI of July and B: NDVI of May. Winter wheat fields are rotated with summer maize, hence they have high NDVI in September and low NDVI in July, denoted by “pink parcels” on the composited NDVI image. Summer crops, such as cotton and maize, always have high a vegetation fraction in July and some crops also have a high vegetation fraction in September, so these fields were denoted by green or yellow parcels in the NDVI composition map. (C, E, G, I, K) are the winter wheat maps of the corresponding NDVI composited images.

Figure 23 shows the spatial distribution of cotton, spring maize and summer maize generated from April 1–August 15 data. Summer maize was the dominant crop in the study area as it was sown after the harvest of winter wheat, while cotton was mainly planted in the southwest of the study area. Although some pixels had missing values due to cloud, the IAIN classifier used in this study could deal with these problems and generate crop maps of high quality. In addition, we only conducted pixel-based classification as the object-segment may introduce some uncertainty for heterogeneous cropland. The results clearly showed the field boundaries, which proves the potential of using Sentinel images for mapping fragmented crop fields.

We then estimated how early we could acquire accurate crop distribution maps. Our result shows that: (1) the classification accuracies of winter wheat is saturated with April 1–May 15 image time series, which is 20 days before harvest time (early June). (2) The classification accuracies of cotton and spring maize were saturated using the April 1–July 15 image time series, which was 4–6 weeks earlier than harvest time. (3) The classification accuracies of summer maize were saturated using the April 1–August 15 time series, which is one month earlier than the summer maize harvest. This study evaluated the potential for early season identification of multiple crops (winter wheat, cotton, spring maize and summer maize) at finer (10 m) spatial resolution than similar existing studies (Hao et al., 2015c; Skakun et al., 2017; Song et al., 2017; Vaudour, Noirot-Cosson & Membrive, 2015). It provides valuable information for crop growth monitoring and management in regions with fragmented crop fields.

## Conclusions

In this study, we proposed an IAIN algorithm to deal with the irregular image time series. Then, we tried to identify major crops in the early season at 10 m resolution and determined how early we could acquire accurate crop distribution maps. The main conclusions are as follows:

1. The IAIN algorithm proposed in this study demonstrated potential to deal with missing values in image time series. The OAs of the classifier for winter wheat and summer crops identification were 99% and 98.55%, respectively. The capability of IAIN to deal with irregular image time series is the basement of early season crop type mapping.
2. In the study area, winter wheat could be accurately identified 20 days before harvest. This was based on April 1–May 15 images, in which the PAs and UAs were greater than 95%. The PAs and UAs of cotton and spring maize were greater than 95% using an April 1–July image time series. This allowed maps to be generated 4–6 weeks earlier than the harvest of those crops. The PAs and UAs of summer maize were above 95% when using image time series longer than April 1–August 15, indicating that summer maize can be accurately mapped more than one month before harvest.

Future studies of early season crop type mapping should analyze further features at finer spatial resolutions (<10 m) to discover new features that can distinguish between crops even earlier. In addition, some new similarity measurements could be used to further IAIN algorithms.

## Supplemental Information

10.7717/peerj.5431/supp-1 Supplemental Information 1 Tables S1–S4. Table S1: JM distance of April ∼ May features. Table S2: Gini importance scores of April ∼ May features. Table S3: JM distance of April ∼ August features. Table S4: Gini importance scores of April ∼ August features.

10.7717/peerj.5431/supp-2 Supplemental Information 2 Training and validation samples.

## Additional Information and Declarations

### Data Availability

The following information was supplied regarding data availability: The raw data are provided in the Supplemental Files.
